# The novel collagen matrikine, endotrophin, is associated with mortality and cardiovascular events in patients with atherosclerosis

**DOI:** 10.1111/joim.13253

**Published:** 2021-05-05

**Authors:** S. Holm Nielsen, A. Edsfeldt, C. Tengryd, H. Gustafsson, A. C. Shore, A. Natali, F. Khan, F. Genovese, E. Bengtsson, M. Karsdal, D. J. Leeming, J. Nilsson, I. Goncalves

**Affiliations:** ^1^ Nordic Bioscience Herlev Denmark; ^2^ Department of Biomedicine and Biotechnology Technical University of Denmark Lyngby Denmark; ^3^ Department of Cardiology Skåne University Hospital Malmö Sweden; ^4^ Department of Clinical Sciences Lund University Malmö Sweden; ^5^ Wallenberg Center for Molecular Medicine Lund University Malmö Sweden; ^6^ Diabetes and Vascular Medicine University of Exeter, Medical School National Institute for Health Research Exeter Clinical Research Facility Exeter UK; ^7^ Department of Clinical and Experimental Medicine University of Pisa Pisa Italy; ^8^ Division of Molecular and Clinical medicine University of Dundee Dundee UK

**Keywords:** atherosclerosis, biomarkers, collagen, endotrophin, extracellular matrix, inflammation

## Abstract

**Background:**

Rupture of atherosclerotic plaques is the major cause of acute cardiovascular events. The biomarker PRO‐C6 measuring Endotrophin, a matrikine of collagen type VI, may provide valuable information detecting subjects in need of intensified strategies for secondary prevention.

**Objective:**

In this study, we evaluate endotrophin in human atherosclerotic plaques and circulating levels of PRO‐C6 in patients with atherosclerosis, to determine the predictive potential of the biomarker.

**Methods:**

Sections from the stenotic human carotid plaques were stained with the PRO‐C6 antibody. PRO‐C6 was measured in serum of patients enrolled in the Carotid Plaque Imagining Project (CPIP) (discovery cohort, *n *= 577) and the innovative medicines initiative surrogate markers for micro‐ and macrovascular hard end‐points for innovative diabetes tools (IMI‐SUMMIT, validation cohort, *n *= 1,378). Median follow‐up was 43 months. Kaplan–Meier curves and log‐rank tests were performed in the discovery cohort. Cox proportional hazard regression analysis (HR with 95% CI) was used in the discovery cohort and binary logistic regression (OR with 95% CI) in the validation cohort.

**Results:**

PRO‐C6 was localized in the core and shoulder of the atherosclerotic plaque. In the discovery cohort, PRO‐C6 independently predicted future cardiovascular events (HR 1.089 [95% CI 1.019 −1.164], *p *= 0.01), cardiovascular death (HR 1.118 [95% CI 1.008 −1.241], *p* = 0.04) and all‐cause death (HR 1.087 [95% CI 1.008 −1.172], *p* = 0.03). In the validation cohort, PRO‐C6 predicted future cardiovascular events (OR 1.063 [95% CI 1.011 −1.117], *p* = 0.017).

**Conclusion:**

PRO‐C6 is present in the atherosclerotic plaque and associated with future cardiovascular events, cardiovascular death and all‐cause mortality in two large prospective cohorts.

## Graphical abstract

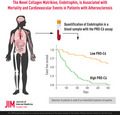

**Abbreviations**
ECM: extracellular matrix
MMP‐11: matrix metalloproteinase‐11
CPIP: Carotid Plaque Imaging Project
IMI‐SUMMIT: innovative medicines initiative surrogate markers for micro‐ and macrovascular hard end‐points for innovative diabetes tools
CRP: C‐reactive protein
LDL: low‐density lipoprotein
HDL: high‐density lipoprotein

## Introduction

Despite recent advancements in treatments for atherosclerotic diseases, the clinical consequences of stroke and myocardial infarction are still the major causes of mortality and morbidity worldwide [1]. There is a need for novel biomarkers which may reflect plaque vulnerability and the risk for recurrent events, and which may help the design of individually tailored treatment strategies [2].

The rupture of an atherosclerotic plaque is the major cause of acute cardiovascular events. These events tend to occur in patients with vulnerable plaques, of the rupture‐prone type, which have a large lipid‐rich core, a thin fibrous cap and an increased inflammatory cell infiltration [3, 4]. A thin fibrous cap is the result of degradation of ECM proteins, especially the main structural ECM proteins, collagen type I, III and VI, which are in the major constituents of the fibrous cap [5]. Collagen integrity is therefore a critical determinant of plaque stability, and disturbed collagen remodelling is a potential marker of plaque vulnerability.

**Fig. 1:**
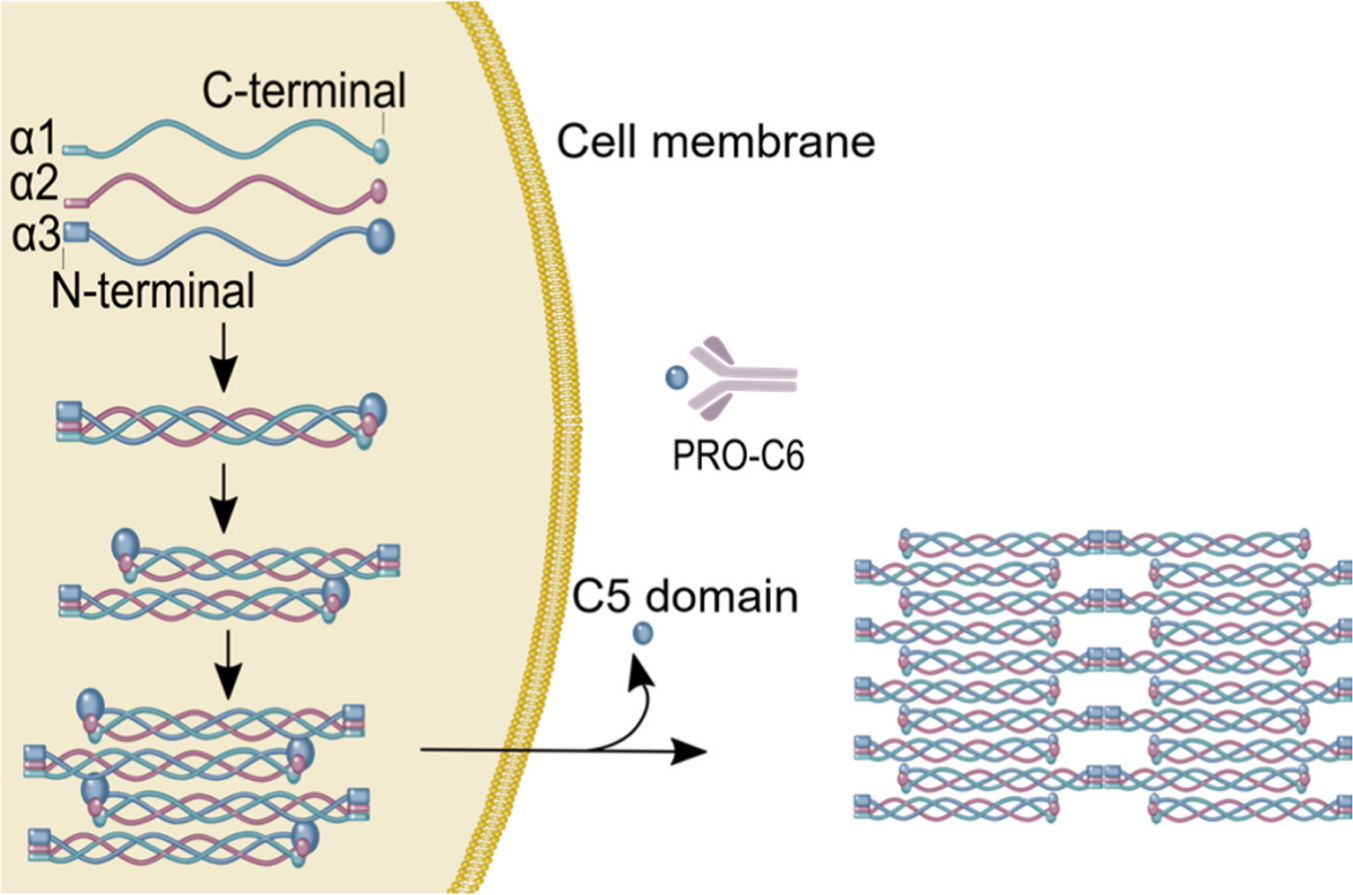
Structure of collagen type Viα3‐chain and release of the pro‐peptide from the α3‐chain. Collagen type VI forms polymers intracellulary before secretion into the ECM. The C5 domain of the α3 chain is immediately cleaved off from the mature collagen type VI tetramer after secretion. The Figure is modified with permission from Karsdal et al. 2019.

Collagen type VI is a beaded filament produced by fibroblasts and is expressed in the atherosclerotic plaque [5]. It is localized in the interface between the interstitial matrix and the basement membrane, where it forms an intricate mesh of microfilaments [6]. Collagen type VI is a heterotrimer composed by the combination of six different α‐chains (α1‐α6), where the C5 domain of the α3 chain has been shown to be important for microfibril formation (Fig. 1) [7]. The C5 domain contains a Kunitz‐type domain which constitutes the matrikine Endotrophin (Fig. 1) [8]. According to a study by Motrescu *et al,* the C5 domain of collagen type VI is a potential substrate for matrix metalloproteinase‐11 (MMP‐11) cleavage [9]. Endotrophin is a bioactive molecule which increases TGF‐β expression by the attraction of macrophages, promoting adipose tissue fibrosis and metabolic dysfunction [8]. The marker PRO‐C6 corresponds to the C‐terminal of the C5 domain which is cleaved off after secretion from the cell, thereby reflecting both collagen type VI extracellular formation and released Endotrophin [10].

The expression of PRO‐C6 in atherosclerotic plaques or the role of PRO‐C6 in predicting cardiovascular events, cardiovascular death and all‐cause death in atherosclerosis patients has not previously been explored.

In this study, PRO‐C6 was detected with immunohistochemistry in advanced human carotid atherosclerotic plaques and serum PROC‐6 levels were analysed in two clinical cohorts: the Carotid Plaque Imaging Project (CPIP) and the innovative medicines initiative surrogate markers for micro‐ and macrovascular hard end‐points for innovative diabetes tools (IMI‐SUMMIT).

## Methods

A more detailed method description can be found in the online supplementary material.

### Immunohistochemistry

Paraffin sections (6 μm) of the most stenotic part of the human carotid plaques from the discovery cohort (CPIP) were fixed in formalin and used for immunohistochemistry. To stain for PRO‐C6, a monoclonal antibody was used (Nordic Bioscience, Herlev, Denmark) diluted 1:12,000 (0.65 μg/ml). Nuclei counterstaining was performed with Mayer’s haematoxylin. A monoclonal mouse isotype control (ab81032, Abcam, Cambridge, UK) was used in a concentration corresponding to the primary antibody (0.67 μg/ml). Scanning of the stained slides was performed with Aperio ImageScope (version 12.3.2.8013).

### Cohorts

The discovery cohort included 577 patients from the Carotid Plaque Imaging Project (CPIP) cohort, Clinical Research Center (CRC), Lund University, Malmö, Sweden. The validation cohort consisted of 1378 patients from the IMI‐SUMMIT cohort recruited from four different European sites (Dundee, Pisa, Malmö and Exeter University Hospitals). The IMI‐SUMMIT cohort was constructed to study the therapeutic need for new treatments of diabetes complications, such as cardiovascular disease. The cohort was designed to study novel biomarkers that can be used for preclinical and clinical trials and thereby accelerate the drug development. Demographic and clinical data, as well as blood samples, were obtained from both cohorts. Additionally, human carotid atherosclerotic plaque tissue was obtained from the discovery cohort (CPIP). The clinical characteristics of the discovery and validation cohort are shown in Table 1.

**Table 1:** Baseline characteristics of patients in the validation and discovery cohort

	Discovery cohort (CPIP) *n *= 577	Validation cohort (IMI‐SUMMIT) (*n *= 1378)	*p*‐value
Median PRO‐C6 (ng/ml)	7.9 (5.4–10.3)	8.9 (7.2–11.4)	2.93 × 10^−15^
Age (years)	72 (66–78)	68 (62–74)	6.66 × 10^−21^
Male (%)	365 (63)	909 (66)	ns
Height (cm, IQR)	171 (164–177)	170 (164–176)	ns
Weight (kg, IQR)	78 (68–88)	84 (74–94)	4.01 × 10^−16^
BMI (IQR)	26.4 (24.0–29.3)	28.6 (25.7–32.3)	4.14 × 10^−25^
Waist (cm, IQR)	99 (91–106)	103 (94–111)	3.36 × 10^−9^
Diabetes (%)	132 (23)	917 (66)	3.13 × 10^−72^
Hypertension (%)	437 (76)	924 (67)	1.25 × 10^−4^
Obesity (%)	119 (21)	539 (33)	3.55 × 10^−18^
Statins (%)	523 (91)	915 (67)	2.95 × 10^−31^
Current smoking (%)	190 (33)	146 (11)	4.66 × 10^−30^
Blood samples			
Creatinine (µmol/L)	85 (72–104)	81 (70–93)	4.00 × 10^−6^
CRP (mg/L)	2.7 (1.2–7.0)	1.4 (0.7–3.0)	1.19 × 10^−23^
Total Cholesterol (mmol/L)	4.3 (3.6–5.4)	4.2 (3.6–5.0)	0.04
Triglycerides (mmol/L)	1.3 (1.0–1.7)	1.3 (1.0–1.9)	0.018
LDL (mmol/L)	2.6 (1.9–3.5)	2.3 (1.8–3.0)	3.89 × 10^−8^
HDL (mmol/L)	1.2 (1.0–1.5)	1.3 (1.0–1.5)	4.00 × 10^−5^
HbA1c (mmol/mol)^a^	41 (37–48)	48 (40–59)	5.55 × 10^−18^

Values are median, interquartile range (IQR) or *n* (%). For comparison between the discovery cohort and the validation cohort, Mann–Whitney U‐tests were used for continuous variables and chi‐square tests were used for categorical variables. *p* < 0.05 were considered significant.

^a^ HbA1c only for subjects with diabetes in the discovery cohort (CPIP).

### Discovery cohort ‐ CPIP

Five hundred and seventy‐seven patients who underwent carotid endarterectomy at the Vascular Department at Skåne University Hospital (Malmö, Sweden) between 2005 and 2017 participated in the study. Indications for surgery were ipsilateral symptoms (amaurosis fugax, transitory ischaemic attack or stroke) along with a degree of stenosis > 70%. Surgical indications for asymptomatic patients were stenosis > 80%. Eight of the patients were included on two occasions as they underwent surgery at two occasions. All patients were assessed by a neurologist preoperatively. Clinical data and cardiovascular risk factors such as age, hypertension, smoking, obesity, statins and family history of cardiovascular events were recorded in a patient survey at time of inclusion. Blood levels of C‐reactive protein (CRP), low‐density lipoprotein (LDL), high‐density lipoprotein (HDL), triglycerides and creatinine were obtained from medical charts. The carotid plaques were taken from surgery, snap‐frozen in liquid nitrogen and stored at −80°C. Serum samples were collected 24 hours before surgery and processed immediately according to standard protocols. Informed consent was given by all patients, and the study was accepted by the local ethics committee (472/2005).

### Validation cohort – IMI‐SUMMIT

The IMI‐SUMMIT study cohort was used as a validation cohort. This study included four groups: patients with type 2 diabetes (T2D) and cardiovascular disease (CVD), patients with T2D and no CVD, patients with CVD but no diabetes and patients without diabetes or CVD. Exclusion criteria included renal replacement therapy, malignancy requiring active treatment, end‐stage renal disease, any chronic inflammatory disease on therapy, previous bilateral carotid artery invasive interventions or atrial fibrillation. Demographics and clinical characteristics, including medication and physical and laboratory examinations, were obtained according to a predefined study protocol at all four participating centres. The variable previous CVD included nonfatal acute myocardial infarction, hospitalized unstable angina, resuscitated cardiac arrest, any coronary revascularization procedure, nonfatal stroke, transient ischaemic attack confirmed by a specialist, lower extremity artery disease defined as an ankle‐brachial pressure index (ABPI), less than 0.9 with intermittent claudication, or prior corrective surgery, angioplasty or above‐ankle amputation. Diabetes was defined based on contemporary or historical evidence of hyperglycaemia (according to World Health Organization 1998 criteria; fasting plasma glucose 7.0 mmol/L or 2‐h plasma glucose 11.1 mmol/L, or both) or by current medication with insulin, sulphonylureas, metformin or other antidiabetic drugs. Obesity was defined as a BMI above 30. The patients were enrolled at four European University Hospitals (Malmö, Lund University in Sweden, *n *= 399; Dundee University in UK, *n *= 369; Exeter University in UK, *n *= 308; and Pisa University in Italy, *n *= 302) between 2010 and 2013. Informed consent was given by all patients, and the study was accepted by the local ethical committee (2010/464).

### Discovery cohort (CPIP) follow‐up

Follow‐up data from the discovery cohort were available for 433 (75% of cohort) patients until 2015‐12‐31. The operated patients were followed for a median of 43 months. Telephone interviews with patients, medical charts and the Swedish National Patient Register were sources of follow‐up information. Patients operated bilaterally were only followed up regarding their first surgery.

Cardiovascular events included myocardial infarctions, unstable angina, strokes, transient ischaemic attack, amaurosis fugax and any vascular interventions not planned at the time of the operation such as carotid endarterectomy, carotid artery stenting, coronary artery bypass grafting or percutaneous coronary artery intervention, and all deaths with an underlying cardiovascular cause of death. Events or deaths occurring within 72 hours after the endarterectomy were considered procedure‐related and were excluded from the analysis. Events were obtained from the Swedish National Patient Register based upon discharge codes from hospitalization of the patients. The ICD codes used to define cardiovascular events and cardiovascular death can be found in the online supplemental material.

### Validation cohort (IMI‐SUMMIT) follow‐up

Patients in the validation IMI‐SUMMIT cohort were routinely followed up with a new visit after 36 months, and complete follow‐up data were available for 1319 patients (96%). Complete follow‐up data were missing for 59 patients (4.3%) which were not included in the follow‐up analysis. Three end‐points were recorded as follows: all‐cause death, cardiovascular death and cardiovascular events. Cardiovascular events included any fatal or nonfatal cardiovascular events and included all diagnoses used to define cardiovascular disease at baseline described above. The time to events variable was not registered in this study. Therefore, it was not possible to perform Cox proportional hazard regression analysis as in the discovery cohort.

### Biomarker measurements

Serum samples from 557 patients from the discovery cohort and 1378 EDTA plasma samples from the validation cohort were available for measurement of PRO‐C6. PRO‐C6 was measured by a competitive ELISA developed at Nordic Bioscience (Herlev, Denmark). The monoclonal antibody employed in the ELISA assay was raised against the last 10 amino acids of the α3 chain of COL VI (3168’KPGVISVMGT’3177). The antibody was found to be specific to the target sequence when tested against the elongated and truncated peptide, published by Sun *et al* in 2015 [11]. The assay has an intravariation of 6.8 % and an intervariation of 9.2%. It has been approved for up to four freeze‐thaw cycles and storage of serum and EDTA plasma samples up to 48 H at 4°C and 20°C as reported previously [11]. The assay was carried out as previously described [11].

### Statistical analysis

Baseline characteristics are described as median and interquartile range (IQR; 25th percentile to 75th percentile) for continuous variables and number (percentages) for categorical variables. Continuous variables were not normally distributed and are therefore presented as median with IQR. Correlations between PRO‐C6 levels and continuous variables were performed using Spearman correlations. Statistical significance was set at *p* < 0.05.

Mann–Whitney U‐test was used for two‐group comparison of PRO‐C6 levels. Survival analysis with Kaplan–Meier curves and log‐rank tests were performed for PRO‐C6 levels divided into tertiles (high, medium and low levels) in the discovery cohort. Cox proportional hazard regression analysis (hazard ratios (HR) with 95% confidence interval, (CI)) was used in the discovery cohort and binary logistic regression (OR with 95% CI) in the validation cohort. Two different regression analyses were used as not all European sites in the validation cohort (IMI‐SUMMIT) had access to equally detailed follow‐up time with the exact date of events. Follow‐up analysis was performed regarding three end‐points: 1) cardiovascular events, 2) cardiovascular death and 3) all‐cause death. The description of the models A, B and C and their creations can be found in the online supplemental material. For statistical analysis, IBM SPSS version 24 was used. Box plots and Kaplan–Meier curves were made using GraphPad Prism version 7.05 (GraphPad Software Inc, CA, USA).

## Results

### PRO‐C6 is expressed in human atherosclerotic plaques

**Fig. 2:**
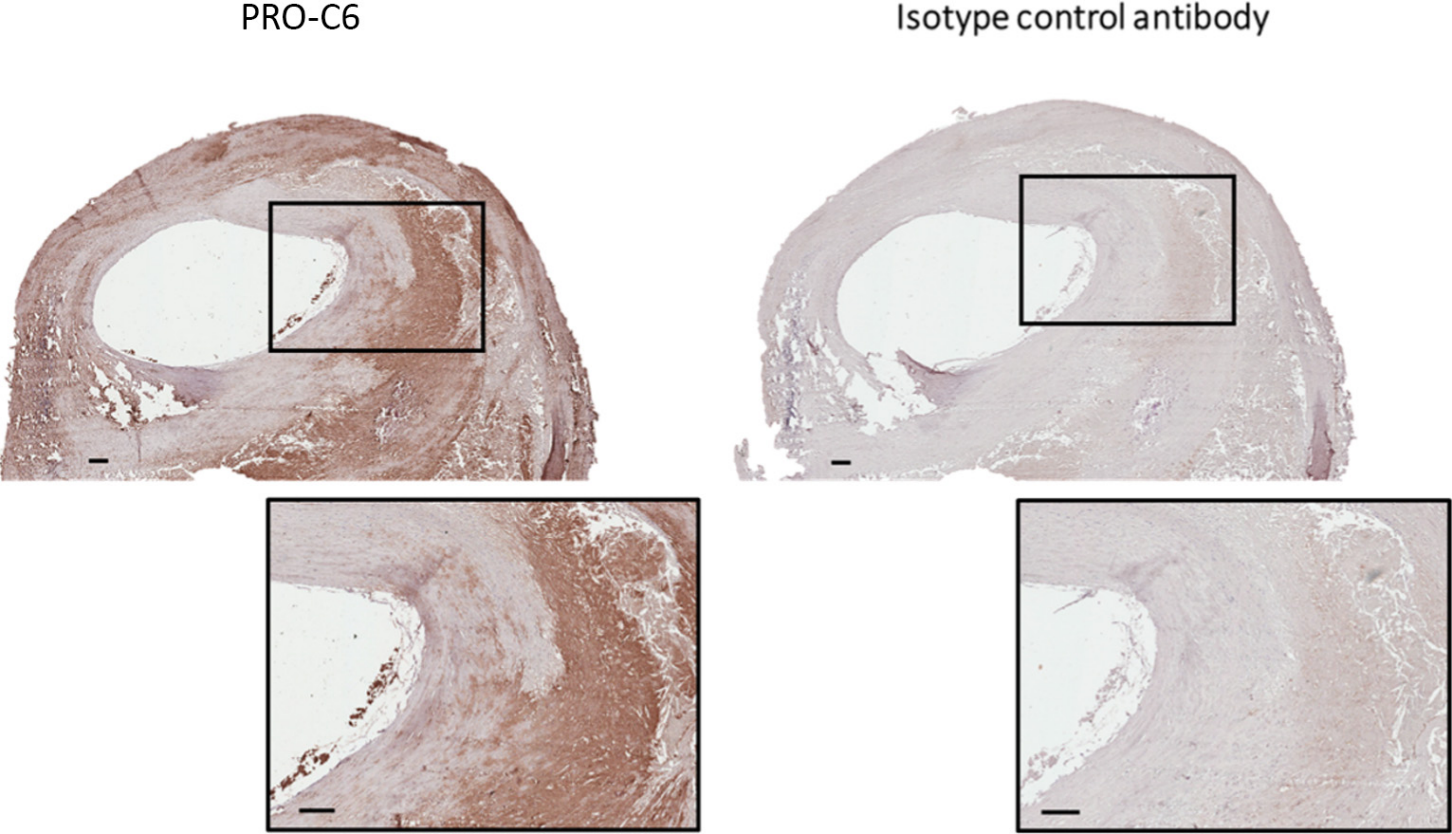
Histological staining of PRO‐C6 in human carotid artery plaque. Brown areas represent immunohistochemistry stainings of PRO‐C6 (left) and staining with an isotype control in matching concentration (right) in a representative advanced atherosclerotic plaque, showing that PRO‐C6 is expressed mostly in the shoulder region and in and around the core. Squares shown in bottom at x2 magnification. Bar represents 200 μm.

PRO‐C6 was expressed in advanced human atherosclerotic plaques and was localized in the core as well as in areas close to the core and in the shoulder regions of the plaque (Fig. 2).

### PRO‐C6 levels and the baseline characteristics of the discovery cohort (CPIP)

The baseline characteristics of the discovery cohort are shown in Table 1. The median plasma level of PRO‐C6 in the discovery cohort was 7.9 ng/ml (IQR, 5.4–10.3).

### PRO‐C6 levels correlate with age, BMI, CRP and creatinine in the discovery cohort (CPIP)

We tested the association of PRO‐C6 levels to risk factors and clinical characteristics in the discovery cohort, particularly considering that PRO‐C6 has previously been linked to progression to end‐stage renal disease [12, 13]. Levels of PRO‐C6 correlated with age (*p* < 0.001), BMI (*p*=<0.05), creatinine (*p*=<0.001) and hs‐CRP (*p*=<0.01, Table 2).

**Table 2:** Spearman correlations for clinical characteristics and clinical blood samples and PRO‐C6 values for 577 patients in the discovery cohort (CPIP) in blue and PRO‐C6 values for 1378 patients in the validation cohort (IMI‐SUMMIT) in black

Discovery cohort (CPIP)		*p*‐value
Clinical characteristics	Spearman's rho	
Age	*r* = 0.312	1.78 × 10^−14^
Waist	*r* = 0.155	8.6 × 10^−4^
Height	*r* = −0.042	ns
Weight	*r* = 0.057	ns
BMI	*r* = 0.104	0.013
Clinical blood measurements		
Creatinine	*r* = 0.285	3.60 × 10^−12^
CRP	*r* = 0.146	5.2 × 10^−4^
Total cholesterol	*r* = −0.006	ns
Triglycerides	*r* = 0.076	ns
LDL	*r* = 0.009	ns
HDL	*r* = −0.039	ns
HbA1c^a^	*r* = 0.015	ns

Validation cohort (IMI‐SUMMIT)		*p*‐value
Clinical characteristics	Spearman's rho	
Age	*r* = 0.181	1.35 × 10^−11^
Waist	*r* = 0.130	2.0 × 10^−6^
Height	*r* = −0.130	ns
Weight	*r* = −0.086	0.001
BMI	*r* = 0.114	2.5 × 10^−5^
Clinical blood measurements		
Creatinine	*r* = 0.273	2.58 × 10^−24^
CRP	*r* = 0.137	3.88 × 10^−7^
Total cholesterol	*r* = −0.046	ns
Triglycerides	*r* = 0.061	0.025
LDL	*r* = −0.038	ns
HDL	*r* = −0.092	6.6 × 10^−4^
HbA1c^a^	*r* = 0.110	5.2 × 10^−5^

Abbreviations: BMI, body mass index; CRP, C‐reactive protein; HbA1c, haemoglobin A1c; HDL, high‐density lipoprotein; LDL, low‐density lipoprotein; Ns, not significant.

^a^ HbA1c only for subjects with diabetes in CPIP.

### PRO‐C6 is elevated in patients with preoperative symptoms, diabetes, hypertension, obesity and smoking in the discovery cohort (CPIP)

**Fig. 3:**
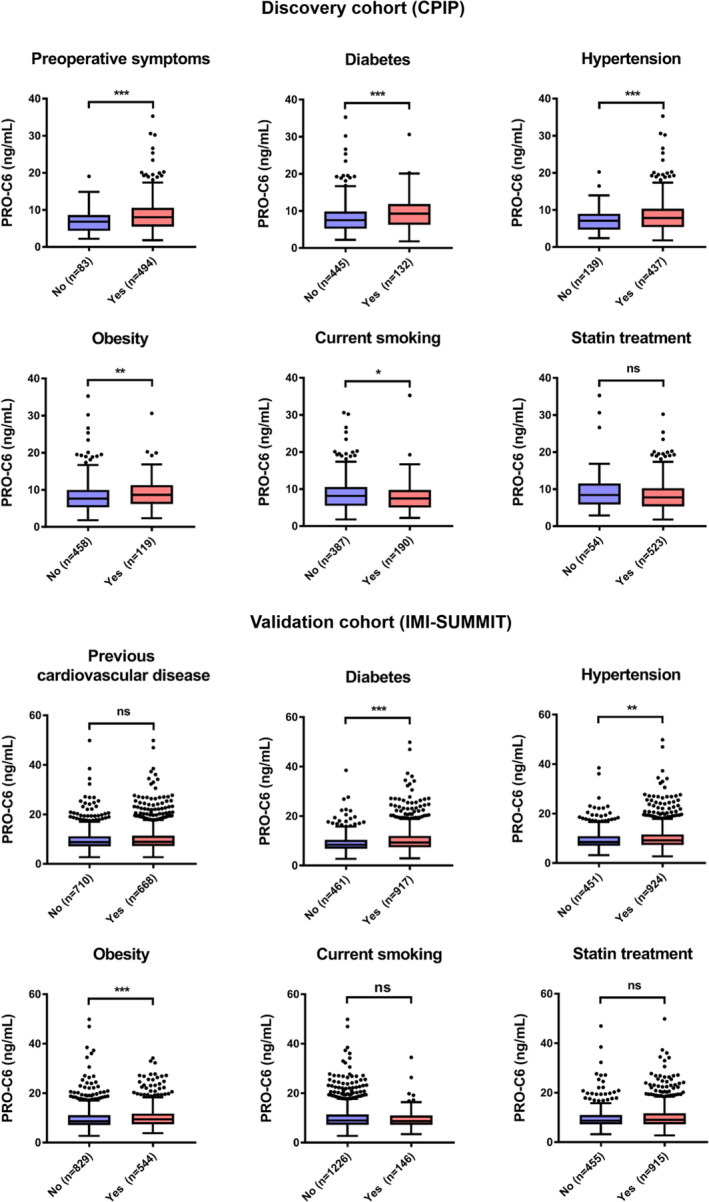
Boxplots illustrating differences in levels of PRO‐C6 in patients with preoperative symptoms/previous cardiovascular disease, diabetes, hypertension current smoking, obsess patients and statin treatment in the discovery cohort (CPIP and validation cohort (IMI‐SUMMIT).). Boxes represent interquartile range (IQR) and whiskers represent 1.5 IQR and dots indicate outliers. Ns = p > 0.05, *p ≤ 0.05, **p ≤ 0.01, ***p ≤ 0.001.

PRO‐C6 levels in patients who had experienced cerebrovascular symptoms preoperatively were significantly higher when compared to those from asymptomatic patients (median (IQR) 8.03 (5.52–10.57) vs 6.84 (4.37–8.64) ng/ml; *p* = 0.001; Fig. 3). Patients with diabetes had higher levels of PRO‐C6 compared with patients without diabetes (9.30 (6.31–11.87) vs 7.53 (5.23–9.86) ng/ml; *p* < 0.001). In patients with hypertension, levels of PRO‐C6 were higher compared with patients without hypertension (8.24 (5.53–10.67) vs 7.10 (4.69–8.99) ng/ml; *p* < 0.001) and obese patient had higher levels compared with nonobese patients (8.27 (5.84–10.48) vs 7.20 (5.07–9.71) ng/ml; *p* < 0.01). Patients who were current smokers had lower levels of PRO‐C6 compared with nonsmokers (7.49 (5.04–9.79) vs 8.16 (5.51–10.57) ng/mL; *p* < 0.05).

### Patients with high levels of PRO‐C6 have an increased risk of all‐cause and cardiovascular death in the discovery cohort (CPIP)

Median follow‐up time for cardiovascular events was 38 months (IQR: 15–59) and 43 months (IQR: 21–65) for death. A total of 77 (17.8%) patients suffered a cardiovascular event, 31 (7.2%) patients died of cardiovascular disease, and 54 (12.5%) patients died of all‐cause death. Patients in the highest tertile of PRO‐C6 had a higher risk of future cardiovascular death as well as all‐cause death compared with the low and medium tertiles combined (Figure S1, log‐rank test *p* = 0.020 and *p* < 0.001, respectively). However, there was no significant association with future cardiovascular events (log‐rank test *p* = 0.23).

In a Cox proportional hazard regression model, levels of PRO‐C6 predicted future cardiovascular events (HR 1.089 [95% CI 1.019 −1.164], *p* = 0.01), cardiovascular death (HR 1.118 [95% CI 1.008 −1.241], *p* = 0.04) and all‐cause death (HR 1.087 [95% CI 1.008 −1.172], *p* = 0.03) after correcting for potential confounders (age, gender, diabetes, hypertension, obesity, smoking, preoperative symptoms, creatinine, hs‐CRP, HDL; Table S1).

### PRO‐C6 levels correlate with established cardiovascular risk factors in the validation cohort (IMI‐SUMMIT)

PRO‐C6 values in the validation cohort correlated with age (*r *= 0.181, *p* < 0.001), waist (*p* < 0.001), BMI (*p* < 0.001) and inversely to height (*p* < 0.01). Levels of PRO‐C6 were also correlated to creatinine (*p* < 0.001), HbA1c (*p* < 0.001) and hs‐CRP (*p* < 0.001) (Table 2).

PRO‐C6 levels were elevated in patients with diabetes compared with patients without diabetes with a median (IQR) 9.3 (7.4–12.0) vs 8.40 (6.80 vs 10.4) ng/ml, *p* < 0.001 (Fig. 3). Levels of PRO‐C6 were higher in patients with hypertension compared with patients without hypertension median (IQR) 9.20 (7.30–11.6) vs 8.5 (7.0–10.9) ng/ml, *p* < 0.01, and obese patients (patients with BMI 30 and above) had higher levels compared with nonobese patients 9.4 (7.4–11.8) vs 8.6 (7.1–11.1) ng/ml, *p* = 0.001.

### PRO‐C6 levels predict cardiovascular events in the validation cohort (IMI‐SUMMIT)

A total of 42 patients (3.2%) died during the 3‐year follow‐up time, and of those, 13 patients (31%) died of cardiovascular causes. A total of 145 (11%) patients suffered from fatal or nonfatal cardiovascular events during the three‐year follow‐up period.

The levels of PRO‐C6 predicted future cardiovascular events (OR 1.063 [95% CI 1.011 −1.117], *p* = 0.017) in a binary logistic regression model which was corrected for potential confounders (age, gender diabetes, hypertension, obesity, smoking, preoperative symptoms creatinine, hs‐CRP, HDL; Table S2). The levels of PRO‐C6 did not predict cardiovascular death but did predict all‐cause death. However, the significance was lost in the last step of correction for confounders.

## Discussion

In this study, we measured a marker of collagen VI formation (PRO‐C6) in subjects with a high prevalence of atherosclerosis from centres across Europe. PRO‐C6 levels in the blood correlated with higher BMI, creatine and CRP and were associated with a higher risk of future cardiovascular events in both the discovery and validation cohort after correcting for potential confounders. PRO‐C6 was associated with a higher risk of cardiovascular and all‐cause death in the discovery cohort. Interestingly, PRO‐C6 was present both in the core area and in the shoulder region of atherosclerotic plaques.

To our knowledge, this is the first study showing that a marker of collagen type VI formation is located in human atherosclerotic plaques and that circulating levels of this marker are able to predict future cardiovascular events, cardiovascular death and all‐cause death.

Noninvasive biomarkers that reflect plaque vulnerability are needed and collagen markers may be an attractive possibility. Collagens are a major constituent of the atherosclerotic plaque, but most research has focused on collagen type I [14, 15, 16]. Nevertheless, there has recently been an increasing interest in collagen type VI in various tissues and diseases [6, 8, 17].

Endotrophin is a cleavage fragment of collagen type VI alpha‐3 chain. Since endotrophin is part of the C‐terminal propeptide of collagen type VI, the PRO‐C6 assay, detecting the ten last amino acids of the alpha‐3 chain may be used to measure endotrophin as well.

Endotrophin is a matrikine that can amplify fibrotic processes, and by amplifying contribute to a thicker cap and a more stable plaque phenotype [8, 12, 18, 19]. Nevertheless, collagen type VI synthesis has showed to be driven by the platelet‐associated platelet‐derived growth factor (PDGF) [20, 21]. Platelets are important contributors to plaque inflammation, and their aggregation to the endothelial surface and activation could be associated with both atherosclerotic plaque formation, as well as acute thrombotic events [22]. Increased levels of released endotrophin could therefore be associated with an increased endothelial platelet aggregation. Other matrikines have previously been described as proteolytically released bioactive ECM fragments [23]. One example of this is Tumstatin, which is an MMP‐9‐derived fragment from the alpha‐3 chain of collagen type IV known to inhibit angiogenesis [23]. This suggests that ECM‐generated matrikines, such as endotrophin, may have a biological role in ECM remodelling, potentially affecting plaque vulnerability [10].

PRO‐C6 levels were higher in subjects with diabetes compared with nondiabetics: PRO‐C6 has previously been identified as a predictive biomarker of treatment response in type 2 diabetes [21]. Moreover, PRO‐C6 has also previously been shown to be a marker for all‐cause mortality in patients with type 1 and type 2 diabetes with kidney damage, and in patients with chronic kidney disease [13, 17, 21].

In the discovery and validation cohort, levels of PRO‐C6 correlated with age and increased creatinine, indicating that collagen VI formation might increase with age and worsened kidney function. The correlations seen in both cohorts between levels of PRO‐C6 and markers of metabolic dysfunction, BMI, waist and CRP further strengthen the hypothesis that the PRO‐C6 also reflects the matrikine endotrophin, as endotrophin was previously found to be associated to adipose tissue fibrosis, insurgence of insulin resistance and recruitment of inflammatory cells [21, 24]. The lower levels of PRO‐C6 found in current smokers in the discovery cohort were unexpected, however may partly be explained by current smokers in the cohort having a lower weight compared with the nonsmokers (median 75 [IQR 65–87] vs 79 [IQR 69–88] kg, *p* = 0.041). Next to smoking, lower PRO‐C6 levels in the discovery compared with the validation cohort could be due to the higher BMI and waist circumference observed, since endotrophin previously has been associated with adipose tissue fibrosis [8, 24]. The two cohorts in this study have differences in the prevalence of risk factors. There was a lower cardiovascular event rate (11.0 % vs 17.8 %), cardiovascular death rate (1.0 % vs 7.2%) and all‐cause death rate (3.2 % vs 12.5%) in the validation cohort compared with the discovery cohort. The validation cohort study was particularly designed to discover new markers for cardiovascular disease in subjects with diabetes, which explains the larger percentage of patients with diabetes and consequently the higher weight, waist circumference, BMI and Hb1Ac found in the validation cohort. The fact that both cohorts include patients with atherosclerosis, but have differences in their participants, makes them complementary in the evaluation of PRO‐C6 as a clinical biomarker.

Nevertheless, since PRO‐C6 was associated with a higher risk for all‐cause death in both cohorts it seems that PRO‐C6 not only reflects risk for cardiovascular events, but may also reflect other pathological processes that affect the ECM, such as diseases with high tissue turnover^13^, ^21^, ^25^, ^26^.

### Study limitations

PRO‐C6 was detected in the human atherosclerotic plaques, indicating that PRO‐C6 levels in the blood might partly stem from the atherosclerotic plaques in the vasculature. However, the proportion of PRO‐C6 originating from other tissues, such as the adipose tissue, has not been investigated in this study. Furthermore, since this is a prospective study there is a lack of definite proof for causality between levels of PRO‐C6, plaque vulnerability and future cardiovascular events. However, the finding that PRO‐C6 is associated with future events in both these two large prospective cohorts shows that PRO‐C6 is a potential marker to stratify patients at risk for cardiovascular events in a clinical setting. Another limitation is the lack of healthy controls for PRO‐C6 immunohistochemistry; however, this was not possible to obtain for this study.

## Conclusion

This study shows that PRO‐C6 correlates with markers of metabolic dysfunction and cardiovascular risk factors and that PRO‐C6 levels are associated with future cardiovascular events, cardiovascular death and all‐cause death in two different large prospective cohorts.

## Conflict of interests

SHN, FG, MAK and DJL are full‐time employee at Nordic Bioscience A/S. Nordic Bioscience is a privately owned, small–medium‐size enterprise (SME) partly focused on the development of biomarkers. None of the authors received fees, bonuses or other benefits for the work described in the manuscript. FG, MAK and DJL hold stocks in Nordic Bioscience A/S. The remaining authors have nothing to disclose.

## Author contribution

**Hilda Gustafsson:** Methodology (supporting); Resources (supporting).

## Supporting information

**Figure S1.** Kaplan Meier curves for (a) cardiovascular events, (b) cardiovascular death and (c) all‐cause death in the discovery cohort (CPIP).
**Table S1.** Cox regression for continuous values of PRO‐C6 in the discovery cohort (CPIP) for cardiovascular events, cardiovascular death and all‐cause death.
**Table S2.** Binary logistic regression for continuous values of PRO‐C6 in the validation cohort (IMI‐SUMMIT) for cardiovascular events, cardiovascular death and all‐cause death.
**Methods S1.** Supplemental methods.
