# Supplementary material for: The novel collagen matrikine, endotrophin, is associated with mortality and cardiovascular events in patients with atherosclerosis
Source: J Intern Med. 2021 May 5;290(1):179–89. doi: 10.1111/joim.13253 (PMC8359970; doi:10.1111/joim.13253)
Supplement: Supplementary file 1 — Figure S1. Kaplan Meier curves for (a) cardiovascular events, (b) cardiovascular death and (c) all‐cause death in the discovery cohort (CPIP). Table S1. Cox regression for continuous values of PRO‐C6 in the discovery cohort (CPIP) for cardiovascular events, cardiovascular death and all‐cause death. Table S2. Binary logistic regression for continuous values of PRO‐C6 in the validation cohort (IMI‐SUMMIT) for cardiovascular events, cardiovascular death and all‐cause death. Methods S1. Supplemental methods. [file JOIM-290-179-s001.docx]

**SUPPLEMENTAL MATERIAL**

**The Novel Collagen Matrikine, Endotrophin, is Associated with mortality and Cardiovascular Events in Atherosclerotic patients**

Signe Holm Nielsen^1*^, Andreas Edsfeldt^2*^, Christoffer Tengryd^3^, Hilda Gustafsson^3^, Angela C. Shore^4^, Andrea Natali^5^, Faisel Khan^6^, Federica Genovese^1^, Eva Bengtsson^3^, Morten Karsdal^1^, Diana Julie Leeming^1^, Jan Nilsson^3^, Isabel Goncalves^2,3^

^1^Nordic Bioscience, Biomarkers and Research, Herlev, Denmark ^1^Experimental
^2^Department of Cardiology, Skåne University Hospital, Sweden
^3^Cardiovascular Research Unit, Department of Clinical Sciences, Lund University, Malmö, Sweden
^4^Diabetes and Vascular Medicine, University of Exeter, Medical School, National Institute for Health Research Exeter Clinical Research Facility, UK

^5^Department of Clinical and Experimental Medicine, University of Pisa, Pisa, Italy

^6^Division of Molecular and Clinical medicine, University of Dundee, Dundee, UK

^*^: These authors share first authorship

**Running title:** Endotrophin is associated with cardiovascular events and mortality in atherosclerosis

**Correspondence**Signe Holm Nielsen,
Nordic Bioscience A/S
Herlev Hovedgade 207
DK-2730 Herlev

DenmarkE-mail: shn@nordicbio.com

**Supplemental Figures**

**Supplemental Figure 1. Kaplan Meier curves for A) cardiovascular events, B) cardiovascular death and C) all-cause death in the discovery cohort (CPIP).** The red lines represent patients in the highest (3rd) tertile and blue lines represent the lowest and medium tertiles combined (1st and 2nd).

**
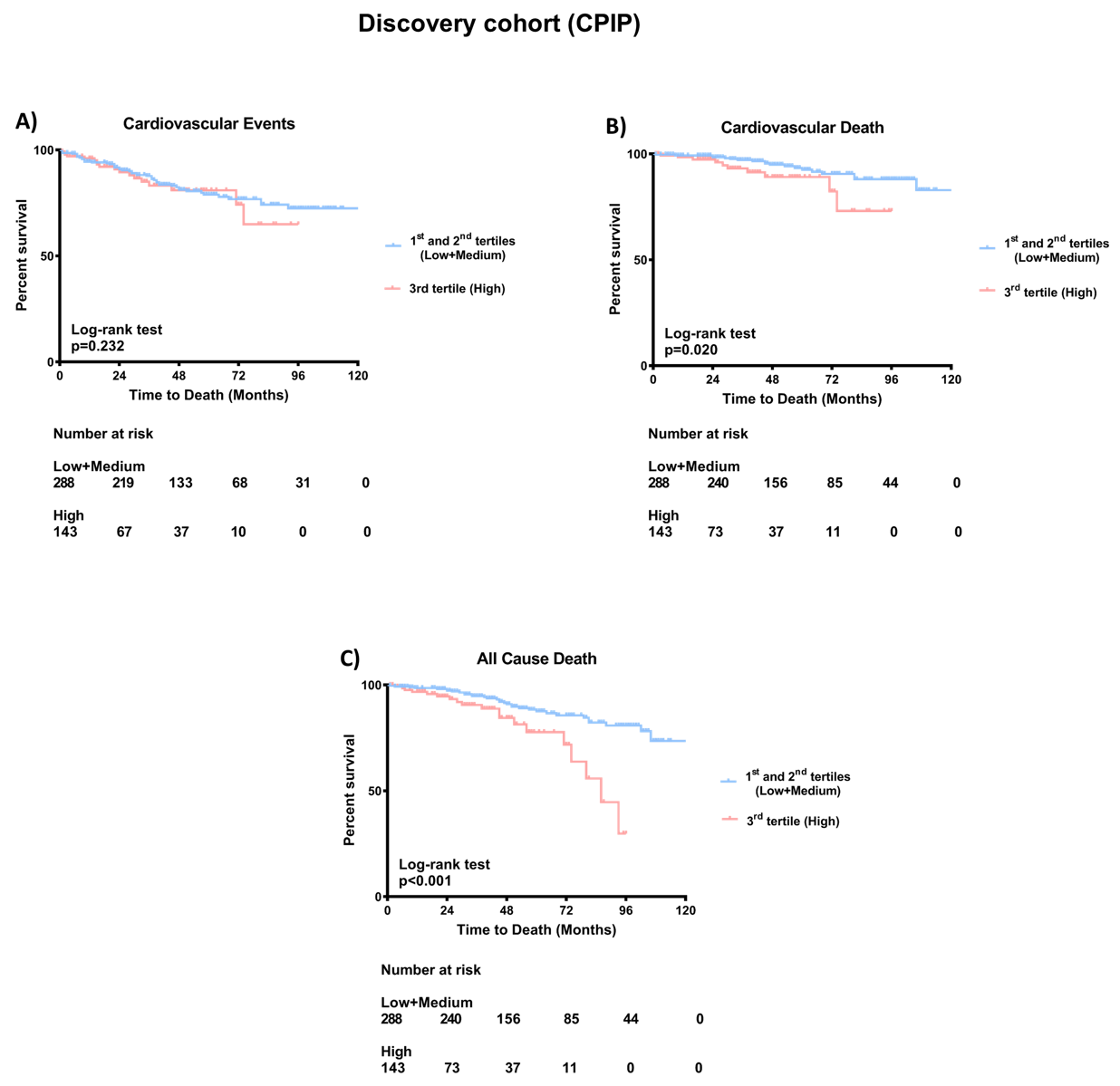
**

**Supplemental Tables**

**Supplemental Table 1. Cox regression for continuous values of PRO-C6 in the discovery cohort (CPIP) for cardiovascular events, cardiovascular death and all-cause death.**

|  | **Discovery cohort (CPIP)** |  |
| --- | --- | --- |
|  | HR (95% CI)* | p-value |
| **Cardiovascular events** |  |  |
| Model A | 1.070 (1.019-1.124) | 7.0x10^-3^ |
| Model B | 1.076 (1.024-1.311) | 4.0x10^-3^ |
| Model C | 1.089 (1.019-1.164) | 0.01 |
|  |  |  |
| **Cardiovascular Death** |  |  |
| Model A | 1.151 (1.080-1.228) | 1.8x10^-5^ |
| Model B | 1.139 (1.067-1.216) | 8.0x10^-5^ |
| Model C | 1.118 (1.008-1.241) | 0.035 |
|  |  |  |
| **All-Cause Death** |  |  |
| Model A | 1.137 (1.079-1.119) | 2.0x10^-6^ |
| Model B | 1.121 (1.063-1.181) | 2.0x10^-5^ |
| Model C | 1.087 (1.008-1.172) | 0.030 |
|  |  |  |

Model A: PRO-C6, Age, Gender.

Model B: PRO-C6, Age, Gender, Diabetes, Hypertension, Obesity, Smoking, Preoperative symptoms.

Model C: PRO-C6, Age, Gender Diabetes, Hypertension, Obesity, Smoking, Preoperative symptoms Creatinine, CRP, HDL.

*per 1 unit change.

**Supplemental Table 2. Binary logistic regression for continuous values of PRO-C6 in the validation cohort (IMI-SUMMIT) for cardiovascular events, cardiovascular death and all-cause death.**

|  | **Validation cohort (IMI-SUMMIT)** |  |
| --- | --- | --- |
|  | OR (95% CI)* | p-value |
| **Cardiovascular events** |  |  |
| Model A | 1.055 (1.023 -1.087) | 5.0x10^-4^ |
| Model B | 1.052 (1.007. -1.099) | 0.020 |
| Model C | 1.063 (1.011 -1.117) | 0.017 |
|  |  |  |
| **Cardiovascular Death** |  |  |
| Model A | 0.623 (0.252. -1.536) | 0.124 |
| Model B | 1.071 (0.971 -1.182) | 0.172 |
| Model C | 1.079 (0.948 -1.229) | 0.284 |
|  |  |  |
| **All Cause Death** |  |  |
| Model A | 1.049 (1.000 -1.101) | 0.048 |
| Model B | 1.072 (1.006 -1.143) | 0.030 |
| Model C | 1.025 (0.938 -1.121) | 0.582 |

Model A: PRO-C6, Age, Gender.

Model B: PRO-C6, Age, Gender, Diabetes, Hypertension, Obesity, Smoking, Previous cardiovascular disease.

Model C: PRO-C6, Age, Gender Diabetes, Hypertension, Obesity, Smoking, Previous cardiovascular disease, Creatinine, CRP, HDL.

*per 1 unit change.

**Supplemental methods**

**Immunohistochemistry**

Paraffin sections (6μm) of the most stenotic part of the human carotid plaques from the discovery cohort (CPIP) were fixed in formalin and used for immunohistochemistry. After de-paraffinization, antigen retrieval was performed in a pH 8.0 Trizma-buffer (Sigma-Aldrich, Stockholm, Sweden) for 15 minutes at 100^o^C. Blocking was performed with 10% bovine serum albumin (BSA) in tris-buffered saline (TBS) for 30 minutes. To stain for PRO-C6 a monoclonal antibody was used (Nordic Bioscience, Herlev, Denmark) diluted 1:12,000 (0.65 μg/mL) in TBS with 1% BSA in TBS and incubated overnight at 4^o^C. MACH3 probe and horseradish peroxidase (HRP) polymer (Biocare Medical, Pacheco, CA, USA) were used for detection along with 3,3'-diaminobenzidine (DAB; Vector Laboratories Inc, Burlingame, CA, USA). Counterstaining of nuclei was performed with Mayer’s hematoxylin. A monoclonal mouse isotype control (ab81032, Abcam, Cambridge, UK) was used in a concentration corresponding to the primary antibody (0.67 μg/ml). Scanning of the stained slides was performed with Aperio ImageScope (version 12.3.2.8013).

**Cohorts**

The two study cohorts included a total of 1,955 patients. The discovery cohort included 577 patients from the Carotid Plaque Imaging Project (CPIP) cohort, Clinical Research Center (CRC), Lund University, Malmö, Sweden. The validation cohort consisted of 1,378 patients from the IMI-SUMMIT cohort recruited from four different European sites (Dundee, Pisa, Malmö and Exeter University Hospitals). The IMI-SUMMIT cohort was constructed to study therapeutic need for new treatments for diabetes complications, such as cardiovascular disease. The cohort was designed to study novel biomarkers, that can be used for preclinical and clinical trials and thereby accelerate the development of drugs. Demographic and clinical data, as well as blood samples were obtained from both cohorts. Additionally, human carotid atherosclerotic plaque tissue was obtained from the discovery cohort (CPIP). The clinical characteristics of the discovery and validation cohort are shown in Table 1.

**Discovery cohort - CPIP**

Five hundred and seventy-seven patients who underwent carotid endarterectomy at the Vascular Department at Skåne University Hospital (Malmö, Sweden) between 2005 and 2017 were included to participate in the study. Indications for surgery were ipsilateral symptoms (amaurosis fugax, transitory ischemic attack or stroke) along with a degree of stenosis >70%. Surgical indications for asymptomatic patients were stenosis >80%. Eight of the patients were included at two occasions as they underwent surgery at two occasions. All patients were assessed by a neurologist pre-operatively. Clinical data and cardiovascular risk factors such as age, hypertension, smoking, obesity, statins, and family history of cardiovascular events were recorded in a patient survey at time of inclusion. Blood levels of C-reactive protein (CRP), low density lipoprotein (LDL), high density lipoprotein (HDL), triglycerides and creatinine were obtained from medical charts. The carotid plaques were taken from surgery, snap frozen in liquid nitrogen and stored at -80^o^C. Serum samples were collected 24 hours before surgery and processed immediately according to standard protocols. Informed consent was given by all patients and the study was accepted by the local ethics committee (472/2005).

**Validation cohort – IMI-SUMMIT**

The IMI-SUMMIT study cohort was used as a validation cohort. This study included four groups: patients with type 2 diabetes (T2D) and cardiovascular disease (CVD); patients with T2D and no CVD; patients with CVD but no diabetes and patients without diabetes or CVD. Exclusion criteria included renal replacement therapy, malignancy requiring active treatment, end-stage renal disease, any chronic inflammatory disease on therapy, previous bilateral carotid artery invasive interventions, or atrial fibrillation. Demographics and clinical characteristics, including medication and physical and laboratory examinations, were obtained according to a predefined study protocol at all four participating centres. The variable previous CVD included nonfatal acute myocardial infarction, hospitalized unstable angina, resuscitated cardiac arrest, any coronary revascularization procedure, nonfatal stroke, transient ischemic attack confirmed by a specialist, lower extremity artery disease defined as an ankle-brachial pressure index (ABPI), less than 0.9 with intermittent claudication, or prior corrective surgery, angioplasty, or above-ankle amputation. Diabetes was defined based on contemporary or historical evidence of hyperglycemia (according to World Health Organization 1998 criteria; fasting plasma glucose 7.0 mmol/L or 2-h plasma glucose 11.1 mmol/L, or both) or by current medication with insulin, sulphonylureas, metformin, or other antidiabetic drugs. Obesity was defined as a BMI above 30. The patients were enrolled at four European University Hospitals (Malmö, Lund University in Sweden, n=399; Dundee University in UK, n=369, Exeter University in UK, n=308, and Pisa University in Italy, n=302) between 2010 and 2013. Informed consent was given by all patients and the study was accepted by the local ethical committee (2010/464).

**Discovery cohort (CPIP) follow-up**

Follow up data from the discovery cohort was available for 433 (75% of cohort) patients until 2015-12-31. The operated patients were followed for a median of 43 months. Telephone interviews with patients, medical charts and the Swedish National Patient Register were sources of follow-up information. Patients operated bilaterally were only followed up regarding their first surgery.

Cardiovascular events included myocardial infarctions, unstable angina, strokes, transient ischemic attack, amaurosis fugax, and any vascular interventions not planned at the time of the operation such as carotid endarterectomy, carotid artery stenting, coronary artery bypass grafting, or percutaneous coronary artery intervention, and all deaths with an underlying cardiovascular cause of death. Events or deaths occurring within 72 hours after the endartectomy were considered procedure-related and were excluded from the analysis. Events were obtained from the Swedish National Patient Register based upon discharge codes from hospitalization of the patients. The following International Classification of Diseases, Tenth Revision (ICD‐10) codes were used to identify cardiovascular events: G45.9, G45.3, G46, I63.1 to 5, I63.8 to 9, I64.5, I20, I21 to 22, I24.8 to 9, I25.1 to 2, I25.5 to 6, and I25.8. All events were verified by telephone interviews with the patients and by review of the patients’ medical charts.

All deaths were extracted from the Swedish cause of death register from The Swedish National Board of Health and Welfare. Following International Classification of Diseases, Tenth revision (ICD-10) these codes were used for identification of cardiovascular death: I21.9, I25.1, I25.8 to 9, I48, I50.9, I60.9, I61.9, I63.2, I64, I69.4, I71.0, I73.9, I74.9, and I99. The median follow-up for cardiovascular events was 38 (IQR: 15-59) months and 43 (IQR: 21-65) months for death.

**Validation cohort (IMI-SUMMIT) follow-up**

Patients in the validation IMI-SUMMIT cohort were routinely followed up with a new visit after 36 months. Complete follow up data was missing for 59 patients (4.3%) which were not included in the follow up analysis. Three endpoints were recorded: all-cause death, cardiovascular death and cardiovascular events. Cardiovascular events included any fatal or non-fatal cardiovascular events and included all diagnoses used to define cardiovascular disease at baseline described above. The time to events variable was not registered in this study therefore it was not possible to perform Cox proportional hazard regression analysis as in the discovery cohort.

**Biomarker measurements**

Serum samples from 557 patients from the discovery cohort and 1378 EDTA plasma samples from the validation cohort were available for measurement of PRO-C6. PRO-C6 was measured by a competitive ELISA developed at Nordic Bioscience (Herlev, Denmark). The monoclonal antibody employed in the ELISA assay was raised against the last 10 amino acids of the α3 chain of COL VI (3168’KPGVISVMGT’3177)[13]. The assay was carried out as previously described[13]. Briefly, a streptavidin coated 96-well ELISA plate (cat. 11940279; Roche) was coated with the coatingpeptide Biotin-KPGVISVMGT. The plate was washed five times in washing buffer followed by incubation with the standard peptide KPGVISVMGT or sample together with the horseradish peroxidase–conjugated monoclonal PRO-C6 antibody. The plate was washed five times followed by incubation with 3,39,5,59-tetramethylbenzidine (Kem-En-Tec, Taastrup, Denmark) in the dark. To end the reaction, a 1% sulfuric acid solution was added, and the plate was analyzed on the ELISA reader at 450 nm, with 650 nm as the reference. All incubation steps were carried out with constant shaking at 300 rpm.

**Statistical analysis**

Baseline characteristics are described as median and interquartile range (IQR; 25th percentile to 75th percentile) for continuous variables and number (percentages) for categorical variables. Continuous variables were not normally distributed and are therefore presented as median with IQR. Correlations between PRO-C6 levels and continuous variables were performed using Spearman correlations. Statistical significance was set at P<0.05.

Mann-Whitney U test was used for two-group comparison of PRO-C6 levels. Survival analysis with Kaplan-Meier curves and log-rank tests was performed for PRO-C6 levels divided into tertiles (high, medium and low levels) in the discovery cohort. Cox proportional hazard regression analysis (hazard ratios (HR) with 95% confidence interval, (CI)) was used in the discovery cohort and binary logistic regression (OR with 95% CI) in the validation cohort. Two different regression analyses were used as not all European sites in the validation cohort (IMI-SUMMIT) had access to equally detailed follow up time with the exact date of events. Follow up analysis was performed regarding three endpoints 1) cardiovascular events, 2) cardiovascular death and 3) all-cause death. The follow up analyses were corrected for the following potential confounders: age, gender, diabetes, hypertension, obesity, smoking, preoperative symptoms/CVD at baseline, creatinine, CRP, HDL levels. These variables were chosen due to their significant associations with PRO-C6 levels at baseline. Three models were created were Model A only corrected for age and gender. In mode. Model B was corrected for clinical variables that differed (diabetes, hypertension, obesity, smoking, preoperative symptoms) in the clinical characteristics. In the final, Model C, the clinical measurements (creatinine, CRP, HDL) that differed in the clinical characteristics table was also added to the model and corrected for.

For statistical analysis IBM SPSS version 24 was used. Box plots and Kaplan-Meier curves were made using GraphPad Prism Version 7.05 (GraphPad Software Inc, CA, USA).
